# Empathy-Related Responses to Depicted People in Art Works

**DOI:** 10.3389/fpsyg.2017.00228

**Published:** 2017-02-24

**Authors:** Ladislav Kesner, Jiří Horáček

**Affiliations:** ^1^Applied Neurosciences and Brain Imaging, National Institute of Mental HealthKlecany, Czechia; ^2^Department of Art History, Masaryk University BrnoBrno, Czechia

**Keywords:** empathy, art experience, socio-affective processing, affective affordance, esthetic processing, art work

## Abstract

Existing theories of empathic response to visual art works postulate the primacy of automatic embodied reaction to images based on mirror neuron mechanisms. Arguing for a more inclusive concept of empathy-related response and integrating four distinct bodies of literature, we discuss contextual, and personal factors which modulate empathic response to depicted people. We then present an integrative model of empathy-related responses to depicted people in art works. The model assumes that a response to empathy-eliciting figural artworks engages the dynamic interaction of two mutually interlinked sets of processes: socio-affective/cognitive processing, related to the person perception, and esthetic processing, primarily concerned with esthetic appreciation and judgment and attention to non-social aspects of the image. The model predicts that the specific pattern of interaction between empathy-related and esthetic processing is co-determined by several sets of factors: (i) the viewer's individual characteristics, (ii) the context variables (which include various modes of priming by narratives and other images), (iii) multidimensional features of the image, and (iv) aspects of a viewer's response. Finally we propose that the model is implemented by the interaction of functionally connected brain networks involved in socio-cognitive and esthetic processing.

## Introduction

Empathy-related phenomena occupy a central place in contemporary neuropsychology and social and affective neuroscience. This research agenda, spurred both by the rapid spread of neuroimaging, and new conceptual models, has been accompanied in the past two decades by the rediscovery of empathy in the humanities, particularly in art history and theory and in film studies. In itself, this rediscovery can be set within the larger context of what might loosely be termed the “bodily” and the “emotional” turn in the humanities and social sciences (Papoulias and Callard, 2010; Lanzoni, 2012). It is increasingly evident that empathy-related issues offer the possibility for productive interfacing between the sciences of the mind and brain and the humanities. Within the humanities, the role of embodied meaning-making in pictorial and esthetic experience has been examined not only in more philosophical and theoretical writings (e.g., Curtis and Koch, 2009; Coplan and Goldie, 2011) but also in art-historical and critical texts devoted to specific works of art written by some of the leading art historians of our times. Scholars such as Leo Steinberg and Michael Fried have written rich and nuanced accounts of bodily projection and empathic engagements with works of art (Steinberg, 1988, 2001; Fried, 2002). Many works of visual art engage and facilitate complex emotional and empathic reactions, thereby potentially serving as a testing ground for such complex reactions in real-life situations.

The best art-historical accounts of embodiment and emotional engagement with works of art, such as Steinberg's and Fried's, operate on the level of behavioral explanation and are firmly rooted in the phenomenology of their authors' viewing experience, without taking into consideration the current conceptualizations of emotional and empathic reactions. On the other hand, cognitive-psychological and neuroscientific accounts of empathic response to works of art do not, as a rule, incorporate the subjective accounts of actual viewing experiences and/or relevant art-historical facts (Kandel, 2012). However, it is increasingly recognized (see e.g., Bullot and Reber, 2013; psycho-historical framework for empirical esthetics; also Bergeron and Lopes, 2014; Gopnik, 2014) that disregarding the historical and subjective dimensions of artworks, which is typical in the neuroscience of art, actually hinders progress in the field. The specific problem of empathic response to visual artworks spans multiple research fields and requires cross-fertilization among at least four distinct and extensive literatures: (i) the psychology and neuroscience of empathy-related phenomena and the affective processing of visual stimuli; (ii) empirical esthetics/neuroesthetics; (iii) problems of immersion and simulation in fictive worlds; and (iv) relevant art-historical and critical scholarship. In this paper we integrate these literatures in an effort to demonstrate that theoretical models of emotional and empathic response to works of art (which could be used to formulate hypotheses and guide future empirical research) must not be insulated from at least some account of actual experiential engagement with works of art and from art-historical facts. Consequently, after briefly discussing the limits of current models of empathic response to visual art works, we discuss several key modulating factors, focusing primarily on interlinked contextual frames (Section Contextual Framing: Pictorial, Spatial-Experiential, and Cultural Contexts) and the role of the representational medium in staging a “reality effect” (or witness perspective) in empathic response (Section The Role of the Representational Medium and the Reality-Effect in Empathic Response). With this background, we then proceed to outline an integrative model of empathy-related response to figural art works (Section An Integrated Model of Empathy-Related Responses to Figural Artworks).

## The limitations of current models of empathic response to visual art works

Arguably the best-known attempt to link current neuroscientific research on empathy to visual art is that of Freedberg and Gallese (2007). Their theory stems from Gallese's embodied simulation hypothesis, which posits that the mirror-neuron system allows human subjects to directly understand the meaning not only of others' actions but also of their emotions by internally replicating them without any explicit reflective cognition (Gallese, 2003; Gallese et al., 2004). Consequently, Freedberg and Gallese argue against the primacy of cognition in our responses to art. They claim that the “crucial element of esthetic response” involves the activation of universal embodied mechanisms encompassing the simulation of actions, emotions and corporeal sensation, and they conclude: “*Automatic empathetic responses constitute a basic level of response to images and to works of art. Underlying such responses is the process of embodied simulation that enables the direct experiential understanding of the intentional and emotional contents of images. The basic level of reaction to images becomes essential to any understanding of their effectiveness as art*” (Freedberg and Gallese, 2007, p. 202).

Briefly admitting the importance of historical and contextual factors, they insist that they “do not contradict the importance of “basic mechanisms” of response” (ibid; see also Freedberg, 2007; Gallese, 2010). Elsewhere Freedberg claims that the impact of an image such as Rogier van der Weyden's *Descent from the Cross* on both the fifteenth-century and the modern-day viewer would depend on “…a set of cortical responses that have *little to do* with context…but *everything to do* with the connection between sight of the bodies and movements of others and the viewers' sense of their own bodies and movements” (Freedberg, 2011, p. 345–346, emphasis added). Freedberg and Gallese's account apparently simplifies what is a much more complicated process. It has already been criticized on several grounds (Casati and Pignocchi, 2007; Kesner, 2010; Gallagher, 2011; Krois, 2011; Davies, 2014; Schott, 2015; for the most detailed criticism, see Minissale, 2013, p. 84–108), so we shall reiterate only the main point here, which is that the role of embodied simulation (and the mirror-neuron system—hereafter MNS) as the underlying neural mechanism in a viewer's complex understanding of works of art or indeed images is questionable. The authors write: “*viewers report* bodily empathy,” “viewers *often experience*,” “*most spectators* of works of art are familiar with feelings of empathetic engagements with what they see in the work itself” (our emphasis). They argue that embodied simulation is crucial for a direct experiential understanding of images, which is essential to their effectiveness as art. But while we take the first-person accounts of art experience as indispensable source of information, there is little empirical evidence to support such generalizations about viewers' experiences. Moreover, there is scant evidence for the role of MNS in empathic response to art, and most importantly, the empathic response, on our account, cannot be limited to motor resonance with depicted bodies. This is aptly summarized by Minissale: “It could be said that not only does Freedberg overemphasize the importance of empathy in art, but that he also promotes a particularly simplistic form of empathy” (Minissale, 2013, p. 104). While the merits of the simulation theory of empathy and the role of MNS in empathy-related responses to socially salient signals continue to be subjects of intense debate (e.g., Decety, 2010b; Baird et al., 2011; Fan et al., 2011; Gallese and Sinigaglia, 2011; Hickok, 2013; Spaulding, 2013; Caramazza et al., 2014; Mikulan et al., 2014; Ando et al., 2015; Gallese and Caruana, 2016), many authors agree that the MNS does not play a substantive role in understanding emotions and empathic response. As Lamm and Majdandžic (2015, p. 20) recently summarized, “…empathy neither requires, nor can be exhaustively explained by “mirror neurons”. However, it is not our intention here either to dismiss the simulation model of empathy or to deny any role for MNS in the empathic and possibly esthetic experience of art works. Rather, we emphasize that affective resonance heavily depends on a viewer's cognitively elaborated understanding of the depicted person's (and/or the artist's) state of mind, as well as their circumstances, that is, on an extensive imaginative projection, and thus the role of embodied simulation (and MNS as its neural substrate) in reflective empathic experience appears to be rather limited. Consequently, we aim to replace current accounts of (narrowly defined) empathic response to visual arts, which exclusively focus on automatic embodied simulation and MNS activation (Freedberg and Gallese, 2007; Kandel, 2012), with a more inclusive model capable of incorporating empathy-related responses to depicted people within the broader pictorial experience. After all, understanding the power of images to elicit affective and empathic responses in their viewers—a subject of long-standing interest—continues to pose a research challenge with profound social implications.

## Varieties of empathy and the modulatory factors of empathy—a conceptual framework

Given the semantic density of the term “empathy” and the terminological inconsistencies surrounding its usage (among recent reviews, see, e.g., Batson, 2009; Cuff et al., 2014), it is not surprising that in discussions of empathy in art, the concept is used to refer to different things—from embodied projection into depicted bodies to feelings of compassion elicited by images. While there is no agreed upon definition of empathy, there is a broad consensus that an evolutionary younger cognitive system is layered on top of affective processes, with the more ancient and more direct visceral-motor mechanism providing scaffolding for more advanced cognitive elaboration and description (Gallese et al., 2004; Keysers and Gazzola, 2009; Shamay-Tsoory et al., 2009; Decety, 2010a, 2011; Fan et al., 2011; Bernhardt and Singer, 2012). Alternatively, empathy-related responses can be conceptualized as involving three domains: motor, affective, and cognitive empathy (Decety and Meyer, 2008). Much attention has been focused lately on the mutual integration and cooperation of the neural structures subserving these domains (Shamay-Tsoory et al., 2009; Cox et al., 2012; Sebastian et al., 2012; Spunt and Lieberman, 2012; Zaki and Ochsner, 2012; Gonzalez-Liencres et al., 2013; Kanske et al., 2015; Mitchell and Phillips, 2015; Schlaffke et al., 2015).

We acknowledge that the response to a work of figural art can in some instances take the form of (largely reflexive) motor resonance with the depicted body or with pictorial elements of the painting (along the lines envisaged in the notion of *Einfühlung*, as originally formulated by Vischer (1873) and Lipps (1903) and as discussed by Freedberg and Gallese (2007), without any further cognitive elaboration. However, here we use the term empathy in a more inclusive sense, as referring to the ability to feel *and* understand what the depicted subject is seen as experiencing. Following a number of influential accounts (e.g., Batson, 1991; Davis, 1996; Decety and Jackson, 2004; Keen, 2006; De Vignemont and Jacob, 2012; Gallagher, 2012; Walter, 2012; Zaki and Ochsner, 2012; Clarke et al., 2015), we take empathy to be more than automatic mirroring and contagion. Instead, it incorporates (i) affective response to a depicted person (sometimes labeled affective empathy), (ii) cognitive understanding, which provides some insight into that person's mental state and situation (sometimes labeled cognitive empathy or affective mentalizing), and (iii) a clear sense of self–other distinction, which precludes the confusing the self with the target. On such an account, empathy involves experiencing in some measure the depicted person's emotional state, that is, it involves: *recognition*, some degree of *understanding* and, in most cases, at least some affective resonance or *sharing* of the inferred mental state. Given the fact that emotional expressions in art works are often intentionally ambiguous and understanding their meaning requires active inference by the viewer, we submit that the affectivity condition (for empathy) is met even when a viewer's response does not match the most plausible interpretation of the depicted state.^1^

The title of the article, “empathy-related responses” is chosen deliberately to embrace both empathy and the closely related phenomenon of compassion or empathic concern, whose distinct behavioral and neuronal profiles have been increasingly acknowledged by some theorists (Bernhardt and Singer, 2012; Klimecki et al., 2013; Singer and Klimecki, 2014). A viewer's reaction to the depicted suffering and misfortune of others can incorporate either self-oriented *empathic distress* (feeling *with* the depicted figures), or other-oriented *compassion* (feeling *for* them), or in most cases, some combination of both components. Importantly for works of visual art, in both aspects the empathic response may in some cases (most frequently in self-portraits) extend from real or imaginary depicted person(s) to the artist, whose presence is either enacted in the image or implied by contextual information.

Empathic response is commonly understood to be strongly modulated both by context and by individual personal characteristics. The main modulating factors, as a number of neuroimaging studies has revealed, include: the intensity of displayed emotion, the appraisal of a situation, the characteristics of the person suffering, attention, the personality traits of the empathizer, previous experience of situations that inflict pain, the degree of attachment to the target, the degree of potential vulnerability or helplessness of an object (Watt, 2005; Gu and Han, 2007; Hein and Singer, 2008; Bernhardt and Singer, 2012; Rameson et al., 2012; Gonzalez-Liencres et al., 2013). The empathic response to visual art works is similarly subject to manifold top-down appraisals and modulations. In what follows we focus on three key factors: (i) *contextual framing*, which can include various modes of priming by narratives and other images and which co-determines patterns of visual exploration of the image; (ii) a unique combination of the *personal dispositions* of the beholder; and (iii) the *characteristics of the image*, including the nature and format of the representational medium.

## Contextual framing: pictorial, spatial-experiential, and cultural contexts

**Figure 1:**
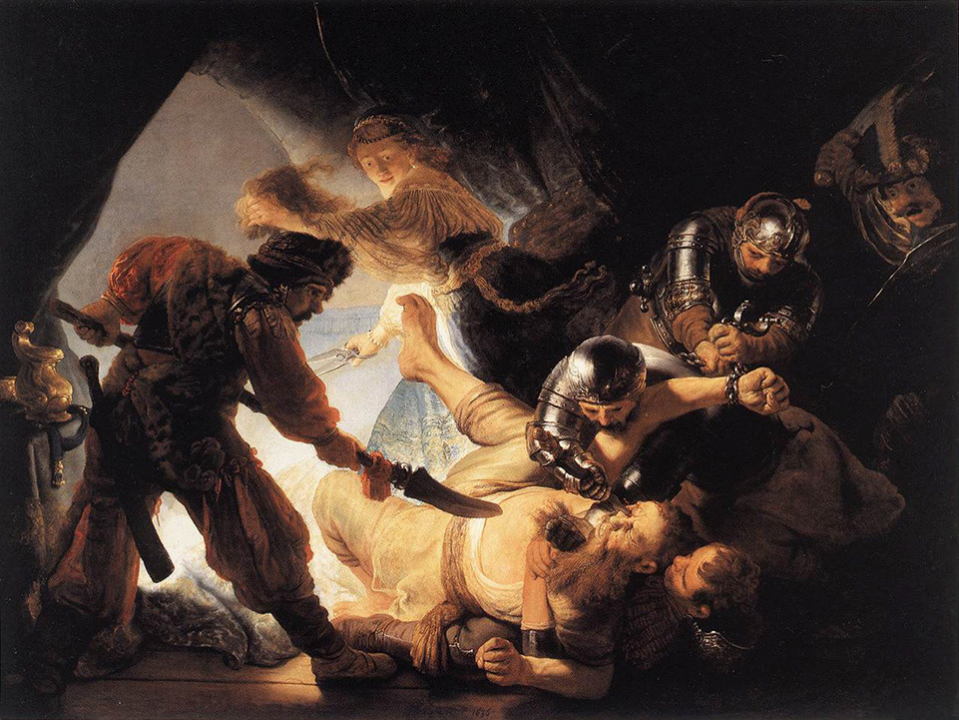
**Rembrandt, *Blinding of Samson*, oil on canvas, 1636**. Image source: Wikimedia Commons library.

The crucial role of context in social cognition generally and empathic response specifically has received increasing attention lately (e.g., Bernhardt and Singer, 2012; Ibañez and Manes, 2012; Melloni et al., 2014). Likewise, the central role of context in art experience is well-established and recent neurocognitive models of art experience highlight the importance of various contextual factors (e.g., Jacobsen, 2006; Bullot and Reber, 2013; Redies, 2015). A number of studies in experimental esthetics have examined various forms of text-based contextual priming (e.g., Millis, 2001; Leder et al., 2006; Smith et al., 2006; Kirk et al., 2009; Noguchi and Murota, 2013; Swami, 2013; Gerger et al., 2014; Silveira et al., 2015), as well as effects of spatial and institutional settings (Brieber et al., 2014, 2015; Krukar, 2014) on art appreciation and experience. For the present purpose, we suggest that three contextual frames must be distinguished: first, the *pictorial context* of the image, i.e., the relationship of the emotionally salient aspects (affective affordances) to the pictorial field or structure as a whole; second, the *spatial and experiential context* of the presentation of the image; and third, the *cultural-social context* of the actual experiential situation, in which the viewer's personal dispositions interact with cultural factors and form expectations. To address the complexities of the contextual priming of empathic response to depicted pain and physical suffering, we now turn to considering a specific example of an art work—*The Blinding of Samson* by Rembrandt from 1,636 (Figure 1). The subject of the painting is the biblical story of the blinding of Samson by the Philistines, which focuses on the cataclysmic effect of agony and on the moment when the actual mutilation takes place. Several authors have highlighted the emotional appeal and effectiveness of the painting. As memorably phrased by art historian Kenneth Clark: “*The Blinding of Samson is an extremely disturbing picture. Only a man of genius could have done anything so consistently horrifying. Apart from the revolting realism of the actual blinding, every detail, every hand and foot, is ugly in itself”* (Clark, 1966, p.20). Other commentators describe the depicted scene as “gory” and as a “drama of extreme brutality” (Perlove and Silver, 2009, p.113), while the author of one of the many monographs on Rembrandt specifically notes that “…in front of this picture we realize the unique power of [Rembrandt's] empathy” (Štech V. V., 1966, p.73).

Recall now the argument that “embodied simulation enables *the direct experiential understanding* of the intentional and emotional contents of images…” (Freedberg and Gallese, 2007, emphasis added). But what does this “direct experiential understanding” amount to in the case of the viewer looking at *Blinding*? We can begin by comparing such a “disturbing and horrifying” image that has the status of an artistic masterpiece to the kind of “emotionally negative” or “aversive” visual stimulus typically used in neuroimaging experiments on pain empathy, such as a naturalistic photograph of a needle being inserted into a hand (Lamm et al., 2007b; Ogino et al., 2007). The sensation of pain inflicted by a needle in a medical procedure will have been endured by most modern-day adults, who then will be able to directly access a vicarious feeling of such a sensation. On the other hand, the physical pain and mental suffering of having one's eyesight violently destroyed is (mercifully) outside the scope of personal experience of almost anyone who encounters *The Blinding of Samson*.

In the presumed empathic resonance with Samson (or the pain of a victim of similar such drastic images, such as Goya's *Disasters of War* or Nicolas Poussin's *Martyrdom*), the viewer can merely evoke a pain caused by whatever trauma to the body or given part of the body (s)he himself has encountered, which then has to be amplified through intentional empathic projection (De Greck et al., 2012) in order to model such unthinkable suffering. But there is obviously more to the depicted scene. Anyone who lost their eyesight through violence would be simultaneously thrust into a mental state of fear, desperation, distress, anger, and possibly many other feelings, that would compound the experience of pain (Price, 2000; Auvray et al., 2010), and this again is something that can only be approximated by the imagination. In fact, Samson's head represents a Gestalt, which encodes both the sensory and the affective components of pain (Kunz et al., 2012) and the effects of his fateful struggle. Thus, even in such persuasive depictions of pain, the experiential understanding, far from being automatic, and direct, appears to involve massive intentional projections by the viewer.

### Pictorial context

A major factor that constrains the nature of the empathic response to Samson's fate has to do with how the most emotionally salient aspects of the scene—what we shall label *affective affordances* (Fuchs and Koch, 2014; Kesner, 2014, see BOX)—are embedded within the overall pictorial space of the painting. In contrast to stimuli typically used in neuroimaging experiments on empathy for pain (e.g., an image showing a limb against the minimal background of a sheet of tissue, exhibiting uniform tonality and no texture, such as in an experiment by Ogino et al., 2007), in Rembrandt's painting, the relative placement of the main affective affordances within the entire pictorial composition assumes critical significance. The crucial details of Samson's head, with a dagger being driven into his right eye and blood and fluids oozing out, comprises roughly 1% of the entire pictorial field. Its imminent context is the struggling body of Samson, composed of a congruent body posture and facial expression. The perception of emotion conveyed by Samson's face is systematically influenced by the emotion expressed by his struggling body (on the contextual factors of the perception of emotional facial expression, see Meeren et al., 2005; Righart and De Gelder, 2008; Barrett et al., 2011; Wieser and Brosch, 2012; Kret et al., 2013).

Box 1 **Affective affordance**

We define **affective affordance** as that component of the image that allows the affective and empathic response to unfold. It is comprised of both the low-level visual properties and the high-level intentional properties of an image. Affective affordances operate on several levels: (i) the level of virtual (depicted) objects and components thereof; (ii) the level of the elements that constitute the representational medium (the image-vehicle)—such as line, brushstrokes, or color; (iii) the level of the overall pictorial structure and composition. The affective salience of human bodies and faces depicted in works of art are always inherently enacted by how the artist works with the artistic medium. In some cases, the artist's intention complicates the activation of affective affordances, whereby the difference between a viewer's response to a real salient object (such as e.g., a face, or a gesture) and their response to a particular depiction of the object constitutes the artwork's esthetic effect (Kesner, 2016). Alongside body postures and gestures, the most important affective (and potentially social) affordances in a visual image are gaze and facial expression and their mutual interaction (see Graham and LaBar, 2012; Rigato and Farroni, 2013 for a review).

In attending to and scanning *The Blinding*, the detail of the head is a strong attractor. Nevertheless, it competes with other points of interest, both at the level of pictorial detail and the composition as a whole. *The Blinding* makes for perceptually an extremely complex scene. To make a quick and incomplete list of the affective and visual affordances vying with Samson's head for attention: on the level of objects, there are the faces and gazes of no fewer than five other figures, as well as their bodies, captured in dynamic movements and gestures, plus a number of other static objects. On the level of pictorial aspects, there is an interweaving of contrasts of light, from dramatic light to deep shadows, often set against each other, the color and texture of the clothes, or the metallic luster of the armor. Finally, there is the effect of the entire spatial setting of the composition, which both integrates the individual virtual objects depicted and addresses the viewer with its own dynamism. The composition enacts a dense interrelationship between the human figures depicted: on a phenomenological level, scanning away from Samson's agony to other figures and faces and/or other visual-semantic affordances provides a respite from direct engagement with the details of a painful situation (and thus could be described as down-regulating empathic response). At the same time, it is the viewer's unfolding awareness of these other figures' actions in relation to Samson's body that may reactivate empathic response to his suffering.

The active viewer inevitably negotiating his distance from the picture will thus be shifting between emotionally charged areas (affective affordances) and other points of visual interest or saliency. As recent eye-tracking studies have demonstrated, the eye initially tends to fixate on emotional objects rather than on other salient but emotionally neutral ones, and emotional saliency can override visual saliency that is defined by such features as intensity, color, and orientation (Humphrey et al., 2012; Massaro et al., 2012; Niu et al., 2012). The more closely the viewer attends to the picture plane, the greater the likelihood that his attention will be drawn to the surface of the painting, its texture and (other) visually salient features: e.g., the relative intensity of the tone, the distinctiveness or sharpness of the plane edges, optical differences, the way in which the depicted scenes and objects emerge from Rembrandt's handling of the medium. (The interaction between socio-affective/cognitive and esthetic processing will be discussed further in Section An Integrated Model of Empathy-Related Responses to Figural Artworks).

### The spatial-experiential and cultural contexts

**Figure 2:**
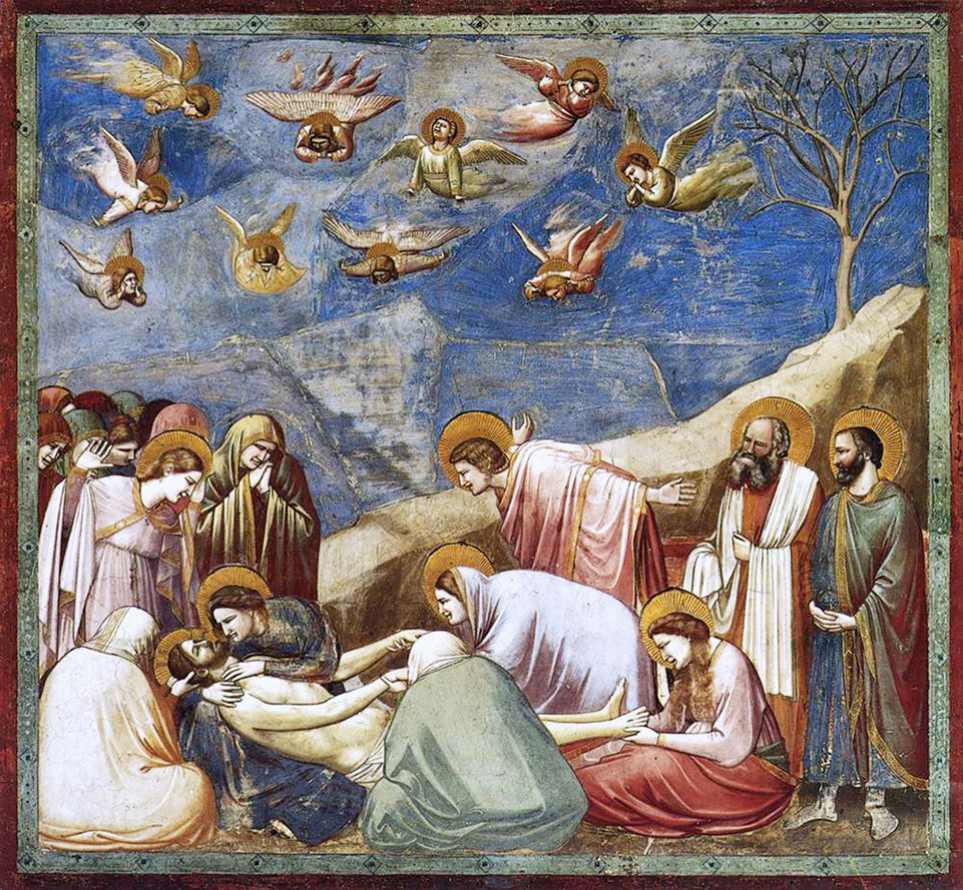
**Giotto di Bondone, *Lamentation of Christ*, Scrovegni chapel, Padua, wall painting, ca 1305**. Image source: Wikimedia Commons library.

The experience of any art work is decisively shaped by the space in which it is presented. The viewer, by actively exercising her art viewing skills within the *spatial/presentational* context, determines and modulates the perception of affective affordances within the picture. The character and nature of empathic response will thus significantly depend on how a person goes about perceptually exploring the art work, involving both the movement of the body in relation to the painting's surface and scanning patterns exercised from a certain point in front of the picture. The viewer's individual pattern of sensory-motor response to an image directly impacts on processes of emotion-regulation and reappraisal, thereby co-determining the nature of the empathic response for the duration of the viewing period and shaping the process by which the picture is interpreted within the framework outlined in section An Integrated Model of Empathy-Related Responses to Figural Artworks. Moreover, the spatial-experiential context significantly shapes the response by providing various means of top-down influence and priming. Emerging evidence points to the role of context-specific information (such as titles of works, labels, and other texts) in making sense and in the appreciation of works of art (Leder et al., 2006; Smith et al., 2006; Kapoula et al., 2009; Silveira et al., 2015). The affective and empathic response to works of art is influenced by texts and narratives on two levels, with semantic information (i) directly guiding the patterns of attention and visual exploration of the work and (ii) providing an overall interpretative and evaluative framework within which the encounter with the work takes place. To provide a specific example, we shall briefly consider another art work—Giotto's *Lamentation* in the Scrovegni Chapel in Padua—as an example of a large category of medieval religious imagery, which can be said to contain a specific “script for action,” for an embodied reaction (Figure 2).

It has been well-established by art historians that scenes of the Passion of Christ, lamentations and other instances of social pain^2^ were efficacious in their original viewing conditions, often eliciting strong emotional responses in their viewers that were manifested in reactions such as weeping, kissing wounds, receiving stigmata, and other forms of embodied response (Belting, 1981; Ringbom, 1984; Freedberg, 1989; Bennett, 2001; Stevenson, 2010). One such account describes the response of Dominican writer Henry Suso's (1295–1366) mother when contemplating images in the Cathedral of Constance during Lent (as quoted in Hamburger, 1998, p. 237): “*From pious contemplation of the worthy suffering of Christ, she felt in an acute manner the great pain that the compassionate mother of God experienced under the cross. And from her sensitivity to this affliction she fell ill in her body, so that she sank to the earth in a dead faint*”. Devotional images were often encountered within a hierotopy (Lidov, 2006)—a multisensory space in which architecture, light, auditory and olfactory sensations all intermingled with the visual experience of images during liturgical ceremonies. Moreover, during communal worship the viewing of images was typically mediated and manipulated by the clergy—by the spoken exegesis of the priest—and even when contemplated privately the viewing of an image was often circumscribed by instructions in psalters, prayer books and paraliturgical compendia, which sometimes included specific instructions on where to direct the gaze (see Hamburger, 1998, p. 80–93). Priming by other sensory impressions and/or spoken or written words thereby functioned to target the gaze and to activate affective affordances within the image. Furthermore, especially in the setting of communal worship, the affective and empathic response was not just primed by words, but also by the socially sanctioned expectations of appropriate emotional display and behavior.

Such modulation and priming amounted to a comprehensive reappraisal and inferences based on historical facts can be thus assessed in the light of contemporary research on affective reappraisal (Wu et al., 2012; Lindquist and Gendron, 2013). Although experimentally verified evidence cannot be provided in the case of past audiences, it is plausible to speculate that semantic priming by words and liturgy may have served to identify and appraise affective affordances, co-determining the exogenous orienting of eye-scanning patterns, attentional allocation, or patterns of glancing vs. prolonged gazing at an image. Taking into account emerging evidence that empathic reaction, particularly the cognitive component, is modulated by the perceived closeness and relationship to the people depicted and affective resonance is limited to close others and extends to outgroup people only with active effort (e.g., Gutsell and Inzlicht, 2012; Eres and Molenberghs, 2013; Meyer et al., 2013), it is furthermore likely that affective resonance served to narrow the empathy gap between the observers and the persons depicted. In sum, priming by spoken or written words and implicit social norms offers an analogy to the regulation strategies used in contemporary experiments: instructions given to subjects to imagine themselves or loved ones in a depicted situation in order to heighten the sense of personal experience (Ochsner et al., 2004; Lamm et al., 2007a; Bebko et al., 2011). Importantly, many instances of contemporary audiences' encounters with pre-modern imagery of social pain are likewise cases of communal experience, in which listening to a live (a companion, a guide, a teacher) or recorded (audioguide) narrative decisively shapes the parameters of the visual encounter and hence how the experience unfolds. To sum up, both the original viewers and the modern-day audience are significantly pre-tuned by mutually dependent factors of their mind-set (participation in the framework of culturally sanctioned actions) and experiential and contextual situation. But crucially, both these factors are constrained by and only unfold through a third factor, namely, the nature of the representational medium that presents the target of the empathic response (as will be discussed in Section The Role of the Representational Medium and the Reality-Effect in Empathic Response).

## Personal factors modulating the empathic response to works of art

An increasing body of evidence points to the crucial role of inter-subjective variation in the profile and the unfolding of both empathy-related responses and art experience. A number of neuroimaging experiments have demonstrated that the empathic response to the pain and emotions of others is modulated by personality traits and affective and cognitive styles (e.g., Avenanti et al., 2009; Calder et al., 2011; Lai et al., 2012). Trait and state anxiety, in particular, has been found to correlate with the magnitude of the response to emotional stimuli (Etkin et al., 2004; McTeague et al., 2011; Ball et al., 2012; Wangelin et al., 2012). At the same time, the crucial importance of art expertise for esthetic processing has been amply documented (e.g., Pihko et al., 2011; Leder et al., 2012; Pang et al., 2013; Else et al., 2015). Based on these and other findings, we suggest that the individual profile of empathic response to a work of art will depend on the interaction of five factors: first, the *individual characteristics* of the subject (including his/her age, gender, parenthood, his/her role as caregiver etc., and previous experience with depicted pain or misfortune); second, the subject's *dispositional empathy* (Davis, 1983), defined as the viewer's responsiveness to the observed experiences of others, has been found to correlate with the frequency and magnitude of empathic response (Davis, 1996; Avenanti et al., 2009); third, the subject's *cultural-cognitive competence* in relation to the experiential situation; fourth, the *intergroup empathy bias*, that is, the perceived closeness and relationship to the person or people depicted; and fifth, the *momentary psychosomatic state* of the observer. The critical among these variables are dispositional empathy and cultural-cognitive competence.

The culture-cognitive competence, which we take to be broader in scope than “expertise,” incorporating experience, skills and knowledge related to viewing art works and making sense of cultural products, may be the most important personal characteristic. Both the viewing of art and empathy (in some descriptions) are a type of skill, and in the experience of art they mutually reinforce each other. Importantly, higher cultural-cognitive competence (Kesner, 2006) need not correlate with a stronger empathic response and may in fact prove constraining and inhibit the empathic response. The viewer's knowledge that (s)he is looking at a masterpiece by an acknowledged artistic genius and her culturally-ingrained expectations vis-à-vis such a great work of art—for example, that one's experience should primarily be one of wonder at and appreciation of the artistic accomplishment, rather than a direct, bodily-mediated reaction—will likely affect the patterns of scanning and attentional allocation (e.g., prioritizing visual-semantic saliences related to esthetic meaning over affective affordances), thus circumventing and down-regulating the development of imminent, bodily-mediated reactions. In a similar vein, if the viewer's mind is focusing on an art-historical interpretation of the intended message of the painting (e.g., to offer an insight into biblical metaphor when Samson's suffering is to be understood as opening the path to his redemption, and blindness becomes a prerequisite for spiritual vision; see Perlove and Silver, 2009, p.113) this semantic knowledge may strongly modulate the empathic reaction in the sense of a down-regulation and reappraisal of affective feelings for Samson as the victim of horrendous mutilation. In an optimal scenario, the developed skills of viewing and making sense of art allow a flexibility of response response, which can range between empathy-related and esthetic aspects of experience (as we detail in section An Integrated Model of Empathy-Related Responses to Figural Artworks).

## The role of the representational medium and the reality-effect in empathic response

Current accounts of the empathic response to art do not sufficiently consider the crucial role played by the mediating effect of the representational medium. However, any comprehensive account of empathic response to art needs to take into account how the contextual and relational modulation of empathic response is realized vis-à-vis the interaction of the viewer with the particular medium of representation. To begin with, there is an obvious difference between the response to an observed real event unfolding before the subject and a depicted event.^3^ Observing representation provides a different set of affordances for the viewer's engagement than being present at the scene of real-life suffering. Consequently, every visual image modulates the empathic response by the simple fact of its being decoupled from the here and now of actually observing an event live, and this is true even in the case of live TV or online broadcasting. Interestingly, this distinction was already taken for granted a long time ago by medieval theologians, who argued that the image might be a more efficient model and stimulus for spiritual movement than the observed behavior of living people (Jezler, 1983).

Perhaps the key question related to our topic here concerns the problem of to what extent the capacity of an artistic or a non-artistic image to elicit an empathic response depends on the viewer's belief in the psychological reality of the depicted event—of the presence of real or fictional characters and the reality of their pain and suffering. This “reality effect” is related to the phenomenon of immersion or absorption in fiction, defined as a vicarious experiencing of events and emotions in fictional representation as if they were real (Walton, 1990; Oatley, 1999). Links between fictional narratives provided by texts, cinema or computer games and empathy have been extensively discussed (e.g., Coplan, 2004; Mar and Oatley, 2008; Mar et al., 2011; Tamir et al., 2016). As Oatley (1999) points out, a prerequisite for the development of empathic skills is emotional transportation into the story. The narrative itself acts to evoke and transform emotions, both directly through the events and characters depicted and (indirectly) through the cueing of emotionally valenced memories. Once evoked by the story, these emotions can in turn influence a person's experience of the narrative (Mar et al., 2011). To be effective, a narrative world has to be real within its context in order to instigate imaginative projections (Green, 2004; Bal and Veltkamp, 2013), that is, the ability to experience the narrative depends on the viewer's subjective feeling of being a witness to the depicted scene. Related evidence suggests that subjects' emotional reactions to unpleasant images as indexed by psychophysiological parameters are attenuated if the subjects perceive the depicted scenes as fictitious instead of real (Mocaiber et al., 2010, 2011). On the other hand, we submit that empathic response does not require that the viewer identify with the depicted character (a detailed discussion of the identification aspect is beyond the scope of this paper, but for a discussion of identification with characters in literature, see Zillmann, 1994; Keen, 2006).

However, the specific role played by different visual media in staging the reality effect or witness perspective for the beholder remains little understood. In some cases, the viewer's implicit belief in the mimetic transparency of the medium elicits in her a subjective sense of being a witness to the depicted scene. Thus, for the observer of medieval images the painting may have had roughly the kind of documentary value that photographic or video-documentary images do for viewers today. Historical evidence suggests that religious images (augmented by narratives) often served to transport the viewer into the time and space of the depicted event or even to change his/her status from that of a *viewer* to that of a *participant* (e.g., Lentes, 2000). Such transient immersion in the fictional world likely intensified the empathic response. To make matters more complicated, however, the adoption of a witness perspective may, or in other instances may not, be related to the degree of verisimilitude (“realism”) with which the medium presents the depicted characters and events. No direct correlation can be established between the phenomenally felt vividness of the depicted event for the viewer and the realism of the image-vehicle. That much is suggested by neuroimaging studies on empathy for pain or social pain that utilized distinctly unnaturalistic stimuli such as computer-generated figures (Masten et al., 2011; Meyer et al., 2013, 2015) or sketches (Krach et al., 2011), yet caused participants to perceive others as active, salient, and significant persons. Other experiments using low-fidelity computer avatars confirm that subjects are capable of interacting with them on phenomenal, behavioral and neuronal levels as if they were real, despite their cognitive knowledge that they are not (Slater et al., 2006; Cheetham et al., 2009). Slater et al.'s observation (2006: p. 6) that the perceptual and neural mechanisms that underlie such a response are largely unexplored still holds true. Thus, manifestly unrealistic (stylized) works of art that proclaim their status as fiction may have the ability to stage a reality effect for a viewer in a particular observational context and trigger an empathic response as effectively as do media that are perceived as providing documentary evidence. On the other hand, there is some evidence that the empathic response to visual stimuli does to some extent depend on the modality through which the image is conveyed. Several studies comparing empathic reactions, measured as the brain activity in subjects observing painful situations in photographs and cartoons, concluded that the neural activity linked to empathy for pain decreased when the reality of the painful stimuli was reduced by presenting painful stimulation in cartoon form (Han et al., 2005; Gu and Han, 2007). Similar results were recently obtained for moving images, where neuronal correlates of emotional empathy were observed in (viewers of) live-action movies, but not in (viewers of) animated movies (Vemuri and Surampudi, 2015). Further elucidating the relationship between the behavioral and neuronal indices of empathic response to the image and the reality effect subjectively perceived by the viewer clearly remains a major goal for future research.

## An integrated model of empathy-related responses to figural artworks

While neuroimaging research on empathy typically focuses on isolating empathic response to visual (or other) stimuli, the empathy-related response to visual art work does not, as a rule, occur in isolation but is embedded within the overall experience or the making sense of an image. Therefore, it is necessary to consider how empathic response is related to the overall experience of the viewer—both in a naturalistic and in an experimental setting. We begin by briefly contrasting a response to a work of art vs. a non-artistic image. Empathic concern and response is generally taken as a precursor and motivation for prosocial, altruistic behavior (Batson, 1991; Preston and de Waal, 2002) and much recent research has focused on investigating the perception-action cascade in empathy (e.g., Mathur et al., 2010; Masten et al., 2011; Zanon et al., 2014). When a person observes non-artistic images (such as media representations) of suffering, the empathic distress elicited by the depicted pain and misfortune of real people may translate into empathic concern and ultimately to some form of prosocial or altruistic behavior: the viewer donates money or tries in some other way to alleviate the ordeal of the depicted victims. Making sense of the image has thus an other-directed prosocial behavior as a tangible output (for a good example of this, see Lieberman, 2013, p. 151–152). On the other hand, when art works are viewed in a museum, this tight coupling of perception and action does not occur, no imminent behavioral reaction to the pain or the negative emotions of the depicted figures is produced. Shaun Gallagher describes the embodied response to art works as an “affordance short-circuit,” which is “. a kinaesthetic-anticipatory response to a non-realizable (non-practical, non-interactionable) affordance, .…an opportunity for experience of the purely possible or maybe even the impossible” (Gallagher, 2011, p. 108–109). How can the embedding of the empathic response within the broader art experience be conceptualized?^4^ We would argue that the experience of empathy-eliciting figural art works prompts a dynamic and fluctuating interaction between two interlinked sets of processes: *socio-affective/cognitive processing*, related to the person perception, which includes one or more domains of empathy and the theory of mind (Blakemore and Frith, 2003; Lieberman, 2007; Ochsner, 2008; Adolphs, 2010; Freeman et al., 2012; Stanley and Adolphs, 2013), and *esthetic processing*, primarily concerned with esthetic appreciation and the judgment of and attention to non-social aspects of the image. Unlike the naturalistic socio-emotional cognition of people, which is typically based on the integration of multimodal cues, (Freeman et al., 2012; Zaki, 2013), here the cues (or affordances) are unimodal, that is, visual. Rather than integrating multimodal cues, an encounter with a figural work of art allows the viewer to focus on the deeper processing offered by a single (visual) modality. In a figural depiction, any affective/social affordance enacted by a painting or sculpture at the same time offers itself as an artistic/esthetic affordance.

### A model of empathy within art experience

**Figure 3:**
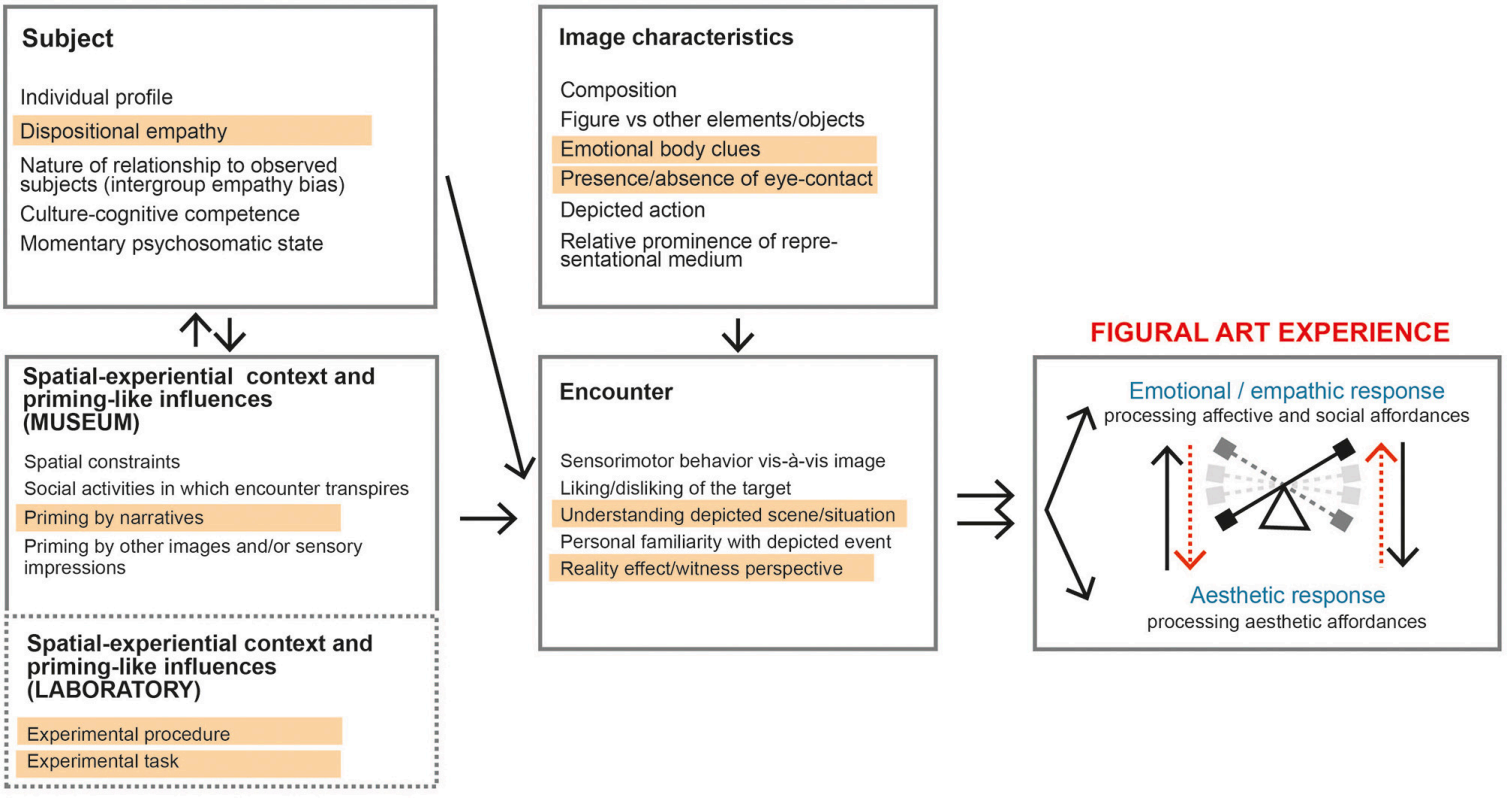
**A model of empathy-related response to figural art work**. Underlined text indicates the critical factors. Double arrows in the rightmost box indicate the bi-directional interaction between socio affective/cognitive and esthetic processing.

Having discussed several critical factors relating to empathic response to figural depiction, we now turn to outlining an integrated model of empathy-related response to figural art work. We present it as a heuristic device that seeks to organize the multiplicity of factors that determine emphatic response and to capture the embedding of empathy-related response within the broader art (pictorial) experience. The model represents—in a necessarily abstract manner—the engagement of a viewer with both *original* figural art work and its *reproduction* in either a private viewing situation or an experimental setting (Figure 3).

**Figure 4:**
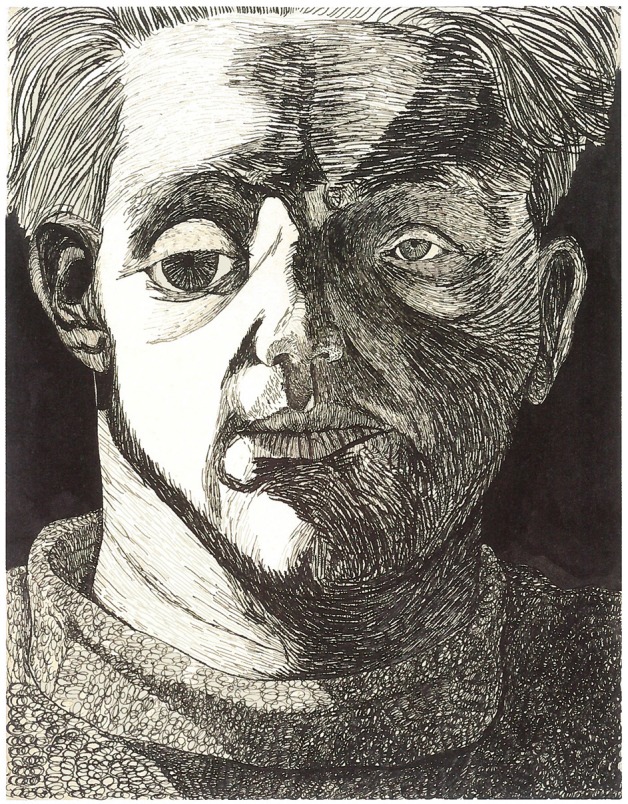
**Zbyněk Sekal, *Self-portrait*, drawing, 1946**. With kind permission of Arbor Vitae Publishers.

The model is envisioned as functioning as follows:

- The viewer, with his or her distinct combination of dispositions (including, most importantly, dispositional empathy and cultural-cognitive competence, see Section Personal Factors Modulating the Empathic Response to Works of Art) engages with a work of art in a specific **spatial-experiential context**. The context modulates how the empathic response unfolds within the overall art experience by creating the spatial constraints of viewing, imposing cultural associations and especially by providing the semantic framework (mostly various forms of top-down priming conditions) of the perceptual encounter (as discussed above Section Contextual Framing: Pictorial, Spatial-Experiential, and Cultural Contexts).
- The specific pattern of interaction (i) between different empathy-related mechanisms and (ii) between socio-affective/cognitive and esthetic processing as the experience unfolds is simultaneously co-determined by the specific **features of the image**. These include: the presentation format, the placement of the figures within the (pictorial) composition (2D images), or the viewing space (3D images), the specific configuration of emotional body clues (gestures, postures), the depicted action, the arousal and valence of emotion embodied by the figure(s), the presence/absence of eye contact with the figure(s), the degree of verisimilitude (realism) and the relative prominence of particular aspects of the representational medium (color, materiality, brushwork etc.). Predictably, other factors being equal, such image characteristics alone may strongly affect the specific patterns of response on a psychological and neural level. Thus, paintings like *Blinding*, with its dynamic composition of figures and its depiction of bodily mutilation, pain and violent action, will be likely to elicit motor empathy and affective pain empathy that are mediated by a pain matrix and action-observation (or mirror) network. On the other hand, as recent experimental evidence suggests, portraits of subjects with emotional expressions who establish eye contact with the viewer /Figure 4/ are more likely to elicit affective empathy and mentalizing (Kesner et al. submitted). Likewise, the relative prominence of the medium at the expense of the realism of the depicted figures may weaken the empathic response. Importantly, all the key variables co-determining the course and magnitude of the empathic response (especially semantic priming, and features of the images) can be experimentally manipulated, thus allowing for empirical testing of the model.
- The relative magnitude of the empathy-related process and the interaction between socio-affective and esthetic processing is from the inception of the perceptual encounter further modulated by the features of the viewer's unfolding response: his/her implicit belief in the reality of the depicted event (or the witness perspective, as discussed in Section The Role of the Representational Medium and the Reality-Effect in Empathic Response), the viewer's like/dislike of the protagonists and understanding of the depicted scene or plot. The interaction between socio-affective/cognitive and esthetic processing as a key aspect of the model is further explored below (Patterns of Interaction between Socio-Affective/Cognitive and Esthetic Processing).
- While none of the factors listed in the diagram in isolation constitutes a necessary or sufficient condition for empathic response to occur, the co-occurrence of key factors will in most cases be a sufficient condition for the empathic response to develop. As a critical feature we single out priming by narratives (or by task/procedure in experimental condition among contextual factors and the presence of highly salient emotional cues in the image, along with direct gaze and effects of the medium that help to enact a witness perspective in a viewer's mind. Depending on their particular combination, the specific pattern of processing ensues, which may take the form of a learned or ritualized “action script” or highly individual, even idiosyncratic response.
- One or more modes of empathic engagements (kinesthetic-motor resonance, affective sharing, cognitive empathy, compassion) depend on and are intertwined with a multitude of other psychological mechanisms, subserving both socio-affective/cognitive and esthetic processing. These involve both reflexive (automatic) and reflective (controlled, volitional, and effortful) processes:

**Table:** 

**Socio-affective/cognitive processing**	**Esthetic processing**
Attentional mechanisms	
Semantic interpretation	
Reappraisal	
Self-reference processing	
Face and body emotional cues processing	Enhanced perceptual processing
Gaze detection	Cue disparity detection
Social categorization	Evaluative judgments/esthetic appraisal
Emotional recognition	Associations generation
	Memory processes
	Episodic simulation
- Finally, while our focus here has been on response to works of art, the model equally applies to response to other empathy-eliciting depictions with non-art status that are circulating in the wider visual culture. For instance, viewing multiple representations of suffering in the media that are primarily intended to communicate information and elicit emotional/empathic reactions in their viewers /Figure 5/ may likewise involve an esthetic appraisal, whereby there occurs an interaction between socio-affective/cognitive and esthetic processing.

**Figure 5:**
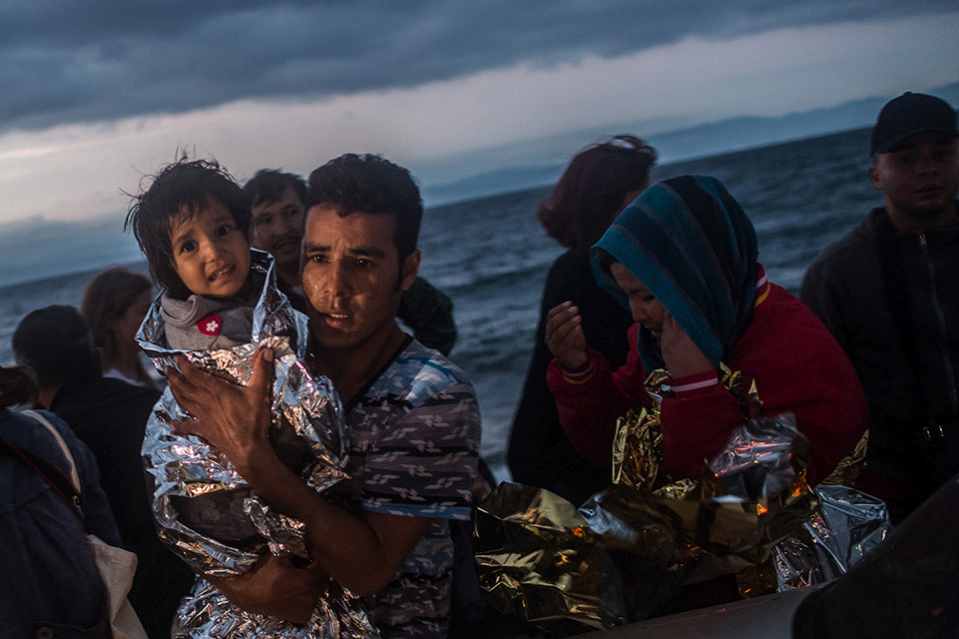
**Filip Singer, from a series *Migration Crisis-Lesbos*, 2015**. With kind permission of the author.

### Patterns of interaction between socio-affective/cognitive and esthetic processing

As described (above), it is always the specific interaction of given image characteristics, personal and contextual factors, as outlined above, that determines the mutual bi-directional interaction between socio-affective/cognitive and esthetic processes for the duration of the encounter. In some situations one of the two responses (affective/empathy-related vs. esthetic) may prevail and dominate the experience. An example would be the case of a medieval audience responding emotionally to scenes of the Passion of Christ discussed above, or instances of people talking to portraits, or of people being sexually aroused by statues (Pygmalionism). There is ample (and psychologically relevant) evidence from art history and criticism, anthropology and literature testifying to the occurrence of various affective and sexual responses to depicted persons *as if* they were real. In the case of sculpture in particular the encounter is often intercorporeal, intersubjective and reactive (Stoichita, 2008; Getsy, 2014). What works of art afford is the possibility to switch—within a focused and sustained viewing experience—between the first-person, observational stance and second-person interactive engagement. At the opposite extreme, the experience of images such as *Blinding*, which manifestly solicit an embodied empathic understanding, may in some cases proceed on the level of an appreciation of the work's formal and semantic qualities, without any affective-empathic response. In the case of modern-day viewers, many encounters with empathy-eliciting artworks are probably too brief and superficial for the experience to be able to develop significantly along either trajectory, so that it remains limited to a basic semantic understanding of what the work depicts (as discussed in Kesner, 2014). In the optimal scenario, however, an ongoing encounter with a complex, empathy-eliciting artwork produces a looping or “seesaw” effect, in which the dominance of one aspect gives way to the dominance of another, and they support each other in a mutually reinforcing cycle.

Importantly, our model does not assume that the empathic response develops along any fixed trajectory from affective resonance to cognitively mediated understanding. There is no behavioral evidence for the presumed automaticity of embodied simulation in viewing artworks and no default direction for empathic reaction from (what embodied simulation theorists label) “automatic empathic response as a basic level of response” (Freedberg and Gallese, 2007) to cognitively elaborated empathizing with the situation of the depicted persons should be posited. Observing museum visitors suggests that if anything like an “automatic” or “basic level of” response to a work of art (on the automatic processing in cognition, see Moors and De Houwer, 2006) can be postulated in relation to viewing a work of art, then that response has to do with the perceptual and semantic understanding of the image—recognizing/identifying the subject and conceptually labeling the depicted scene and persons or event. On the contrary, as subjective accounts of experiences suggest, often there is a gradual process of conscious making sense of or coming to terms with the image that extends over a protracted period of viewing, which then opens the possibility for a full-blown empathic response that ultimately incorporates bodily affective resonance, imaginative projection, and feelings of distress and/or sympathy and compassion.

The mutual interaction of socio-affective/cognitive and esthetic processing can proceed in a number of ways, for example (i) when the viewer shifts his/her attention from the depicted human targets to other aspects of the composition (as described above in the case study of *Blinding*)—such attention shifting can be experimentally ascertained by eye-tracking measurements); or (ii) when the viewer encounters depicted faces and bodies that elicit empathy-related responses and are at the same time esthetically appraised as being beautiful or ugly (cf. as quoted above with reference to the *Blinding of Samson*: “*Apart from the revolting realism of the actual blinding, every detail, every hand and foot, is ugly in itself”*.; for an esthetic appraisal of human bodies, cf. Martín-Loeches et al., 2014; Candidi and Aglioti, 2015); or (iii) when the affective/empathic response to the depicted figures and the esthetic appraisal of the picture as a whole proceeds in a bi-directional exchange or continuous loop. For instance, the very formation of an “emotion percept,” which in real-life social vision involves the rapid integration of compound social cues (Adams and Kveraga, 2015; Marchi and Newen, 2015), will in pictorial perception often evolve into an esthetic appraisal that is concerned with detecting and analyzing incongruences between compound social-emotional cues, such as facial expression, gaze, bodily posture, and gesture (such ambiguities often constituting the artistic intention of the image). Once formed, a cognitively elaborated emotion-percept will stimulate further esthetic appraisal, or it will trigger an empathic resonance in the viewer.

Furthermore, in images that present scenes of one or more interacting figures but offer no direct clue as to the nature of their relationship or the meaning of the pictured event, it is mostly the intentional operation of understanding the circumstances of depicted persons as a necessary precondition for affective resonance and embodied empathic understanding (in both its aspects, i.e., feeling *as* and feeling *for* the depicted target). In such cases, it is the viewer's access to some sort of extra-pictorial semantic information and the grasp of the meaning of the depicted situation that both prompts mental simulation and provides clues that lead to a deeper emotional/empathic processing (for a case study of this process in a specific work of art, see Kesner, 2014). Furthermore, as Zillmann (1991) suggested, empathy may be mediated by morally derived affective dispositions toward the target, which again argues against the notion that the empathic response to an image automatically takes precedence Art-historical/critical literature (which, as a rule, continues to be ignored by neuroesthetics research) and literary sources contain a massive amount of revealing evidence of the existence of this kind of “seesaw” effect, a shifting awareness between being confronted with a (depicted) human person and being confronted with an esthetic object (e.g., Herder, 1778; Riegl, 1902; Steinberg, 1988; Stoichita, 2008; Sidlauskas, 2009 to cite some very different examples).

Secondly, in a deep encounter with an empathy-eliciting art work, empathic distress (feeling like the depicted persons) and compassion (feeling for them) will be typically accompanied by a range of mental states that add to the phenomenological complexity of the experience of art. Depending on the particular work of art and the interaction of factors described in the model, (these mental states) may include basic emotions and complex affective states, such as being moved (Hanich et al., 2014), “feeling like crying” (Pelowski, 2015) or morbid fascination (Oosterwijk et al., 2016), but they can also include specific esthetic emotions. Negative emotions and empathic distress, although subjectively felt as something disquieting and distressing, may ultimately be consciously reappraised in positive terms so that the entire experience is remembered as enjoyable (the “enjoyment of tragedy” phenomenon, cf. De Wied et al., 1994) and transformative (Pelowski and Akiba, 2011), hence rewarding and motivating further encounters of this kind. This process of making sense of the image can be productively considered in relation to the constructivist theories of emotional perception and experience (Russell, 2003), and in particular the model of situated conceptualization (Barrett and Satpute, 2013; Barrett, 2015; see also Minissale, 2013: p. 104–06).

## Future challenges

Given the phenomenological and psychological complexity of the empathy-related responses that are an inherent part of art experience and the manifold factors that co-determine the specific course of the experience, two important questions arise: (i) how is the proposed interaction of socio-cognitive and esthetic processing instantiated by brain structures, and (ii) can the model be experimentally verified? The imminent challenge, then, is to develop experimental neuroimaging paradigms for investigating the interaction of socio-affective/cognitive and esthetic processing, under different variables of task conditions/context modulations, image characteristics and personal profiles of subjects.^5^ For instance, our preliminary results from an ongoing pilot study suggest that by emulating semantic priming in naturalistic conditions by providing viewers with specific viewing instructions aimed at guiding their attention either to the subjects and the emotions elicited by the subjects in the observer, or to the representational and artistic features of the work, it is possible to isolate distinct patterns of neuronal activity related to either socio-affective/cognitive or esthetic processing. Furthermore, as it is recognized that the temporal dynamics of the neuronal activity that underlies both affective (empathic) and esthetic experience may be as important as the spatial distribution of the activity (Immordino-Yang et al., 2009; Adolphs, 2010; Cela-Conde and Ayala, 2015; Betti and Aglioti, 2016; Kirsch et al., 2016), a major methodological challenge is to find ways of combining and integrating the data drawn from high spatial resolution (fMRI) with “fast” electrocortical data. Another challenge, to recap what has already been noted, concerns the possibility of experimentally verifying the role of the subjectively perceived reality effect—that is, the degree to which the subject's sense that the depicted figures and events are real (a sense generated both by the characteristics of a representational medium (as such) and by task condition) impacts the magnitude and specific profile of the empathic response.

Finally, the view of empathic response that we embraced here highlights the crucial importance of subjectively felt, conscious experience. As Lamm and Majdandžic (2015) recently pointed out, methods such as fMRI only provide information on neural responses that co-occur with the experience of empathy. While this point may seem self-evident, it is worth emphasizing that any experimental research on empathic response to art that does not attempt to correlate or integrate objective data with first-person reports on subjective experience will provide a greatly limited and constrained view of the phenomenon, such as could be of little interest to scholars outside the neurosciences and certainly would not foster much needed truly interdisciplinary research efforts. We therefore conclude that the greatest general challenge lies in developing experimental paradigms that would permit correlating and integrating data on the neuronal indices of empathic responses to images with behavioral indices and with narrative first-person accounts of empathic response and experience.

## Author contributions

LK developed the structure and main theoretical aspects and has written substantial parts of the paper. JH contributed to the theoretical model and writing of the paper.

### Conflict of interest statement

The authors declare that the research was conducted in the absence of any commercial or financial relationships that could be construed as a potential conflict of interest.

## Footnotes

^1^In discussing “the affectivity condition” (for empathy to occur), Shaun Gallagher highlights the inherent difficulties involved in specifying[determining] the affective state of the observer of a person suffering (Gallagher, 2012, 374–375). We follow Decety and Jackson's (2004, 73) view that an affective response to another person (as one of the principal components of empathy) often, but not always, entails sharing that person's affective state. However, a more comprehensive discussion of this point is beyond the scope of this article.

^2^Here we take the standard definition of social pain as painful feelings arising from social rejection, exclusion, or loss of a loved one (see Eisenberg, 2015 for a review).

^3^In neuroimaging experiments based on “direct observation” of painful stimuli delivered to the bodies of other people or painful social situations affecting other people, the (response) effect invariably occurs when the viewer is presented with photographs, video clips, or other graphic materials, which are intrinsically assumed to be transparent media that straightforwardly present the event as if it were real. In fact, however, such experiments cannot be said to assess the psychological and neuronal dimension of the reaction involved in “witnessing live a painful event” or “directly witnessing a live person,” as they claim to do (Bruneau et al., 2012).

^4^Here we maintain the important distinction between related, yet conceptually distinct notions of art experience and esthetic experience (Palmer et al., 2013). For the purpose of the present discussion we take “esthetic processing” to refer to a range of cognitive and affective mechanisms as detailed below.

^5^We acknowledge, but do not further discuss here, the methodological difficulties of isolating specific socio-affective/cognitive and esthetic processing. As noted by Adolphs (2010, 761), the distinction between social and nonsocial is always introduced by the experimenter.
